# Predicting the Arousal and Valence Values of Emotional States Using Learned, Predesigned, and Deep Visual Features †

**DOI:** 10.3390/s24134398

**Published:** 2024-07-07

**Authors:** Itaf Omar Joudeh, Ana-Maria Cretu, Stéphane Bouchard

**Affiliations:** 1 Department of Computer Science and Engineering, University of Quebec in Outaouais, Gatineau, QC J8Y 3G5, Canada; ana-maria.cretu@uqo.ca; 2 Department of Psychoeducation and Psychology, University of Quebec in Outaouais, Gatineau, QC J8X 3X7, Canada; stephane.bouchard@uqo.ca

**Keywords:** regression, machine learning, cognitive/emotional state, visual features

## Abstract

The cognitive state of a person can be categorized using the circumplex model of emotional states, a continuous model of two dimensions: arousal and valence. The purpose of this research is to select a machine learning model(s) to be integrated into a virtual reality (VR) system that runs cognitive remediation exercises for people with mental health disorders. As such, the prediction of emotional states is essential to customize treatments for those individuals. We exploit the Remote Collaborative and Affective Interactions (RECOLA) database to predict arousal and valence values using machine learning techniques. RECOLA includes audio, video, and physiological recordings of interactions between human participants. To allow learners to focus on the most relevant data, features are extracted from raw data. Such features can be predesigned, learned, or extracted implicitly using deep learners. Our previous work on video recordings focused on predesigned and learned visual features. In this paper, we extend our work onto deep visual features. Our deep visual features are extracted using the MobileNet-v2 convolutional neural network (CNN) that we previously trained on RECOLA’s video frames of full/half faces. As the final purpose of our work is to integrate our solution into a practical VR application using head-mounted displays, we experimented with half faces as a proof of concept. The extracted deep features were then used to predict arousal and valence values via optimizable ensemble regression. We also fused the extracted visual features with the predesigned visual features and predicted arousal and valence values using the combined feature set. In an attempt to enhance our prediction performance, we further fused the predictions of the optimizable ensemble model with the predictions of the MobileNet-v2 model. After decision fusion, we achieved a root mean squared error (RMSE) of 0.1140, a Pearson’s correlation coefficient (PCC) of 0.8000, and a concordance correlation coefficient (CCC) of 0.7868 on arousal predictions. We achieved an RMSE of 0.0790, a PCC of 0.7904, and a CCC of 0.7645 on valence predictions.

## 1. Introduction

The cognitive state of a person can be categorized using the circumplex model of emotional states [1], a continuous model of two dimensions, arousal and valence, where arousal measures the energy level and valence measures the positivity level of a person’s emotion. In this model, emotions are divided into four categories: happy, angry, sad, and relaxed. Each of these emotions is associated with a quadrant of the circumplex model. Happy emotions have a high valence and high arousal, anger a low valence and high arousal, sadness a low valence and low arousal, and relaxed a high valence and low arousal. The arousal and valence values can be estimated via classical or deep machine learning regression. 

We use the RECOLA database [2] which includes audio, video, and physiological recordings of online interactions between human participants to predict arousal and valence values using machine learning techniques. We previously predicted arousal and valence values using the physiological [3,4] and video [4,5] recordings of RECOLA. 

Features are attributes that describe the data. They can be predesigned or learned [6]. Learned features are attributes that are automatically extracted and utilized by deep machine learning solutions during the learning process. On the other hand, predesigned features are attributes that are calculated on the data before the learning process and provided as input to the machine learner. Deep features are another category of predesigned features. Deep features are features that can be extracted from trained deep machine learning models.

Our previous work on the video recordings of RECOLA focused on learned features from convolutional neural networks (CNNs) such as ResNet-18 and MobileNet-v2 using images of full or half faces for the purpose of virtual reality (VR) applications with head-mounted displays covering half of the face of the user [4,5]. For images of full faces [4], MobileNet-v2 achieved a root mean squared error (RMSE) of 0.1220, a Pearson’s correlation coefficient (PCC) of 0.7838, and a concordance correlation coefficient (CCC) of 0.7770 on arousal predictions. MobileNet-v2 achieved an RMSE of 0.0823, a PCC of 0.7789, and a CCC of 0.7715 on valence predictions from images of full faces. For images of half faces [5], MobileNet-v2 achieved an RMSE of 0.1495, a PCC of 0.6387, and a CCC of 0.6081 on arousal predictions. MobileNet-v2 achieved an RMSE of 0.0996, a PCC of 0.6453, and a CCC of 0.6232 on valence predictions from images of half faces. We later expanded our work to analyze and assess the predesigned visual features, extracted from the video recordings of RECOLA [7]. In [7], we proposed a novel combination of processing steps to prepare the predesigned visual features for regression. We leveraged machine learning solutions such as regression trees, kernel regression, and ensemble regressors to predict the arousal and valence values of cognitive states. As a result, we achieved our best performance of an RMSE of 0.1033, a PCC of 0.8498, and a CCC of 0.8001 on arousal predictions. We achieved an RMSE of 0.07016, a PCC of 0.8473, and a CCC of 0.8053 on valence predictions via an optimizable ensemble model based on bagging and Bayesian optimization. In this paper, we extend our work, from the 10th International Electronic Conference on Sensors and Applications (ECSA-10) [7], by extracting deep visual features using the MobileNet-v2 CNN which was trained and tested in [4,5]. We then apply feature fusion to combine the extracted deep visual features with RECOLA’s predesigned visual features. We then use the extracted deep visual features as well as the combined feature set to perform classical regression for predicting arousal and valence values. To the best of the authors’ knowledge, this combination of predesigned and deep visual features has not been attempted by other researchers who are using RECOLA in their work. Our goal is to identify the model(s) with the best prediction performance to later integrate into a VR system that runs cognitive remediation exercises for users with mental health disorders (e.g., schizophrenia). As such, the prediction of emotional states is important to enable the development of more personalized and effective treatments for those individuals.

Solutions for the prediction of cognitive states, from images of faces, ideally consist of two components: parametrization and the recognition of facial expressions [6]. Parametrization is the process of specifying the visual features and coding schemes to describe the involved facial expressions. The visual features used for the prediction of cognitive states can be appearance or geometric features [8]. Geometric features represent the geometry of the face. Local Gabor Binary Patterns from Three Orthogonal Planes (LGBP-TOP) [9] is one method that is used in the extraction of appearance features, while facial landmarks [10] are usually used for geometric features. Examples of geometric features include the derivatives of the detected facial landmarks, the speed and direction of motion in facial expressions, the head pose, and the direction of the eye gaze. Appearance features represent the overall texture resulting from the deformation of the neutral facial expression. Appearance features depend on the intensity of an image, whereas geometrical features determine distances, deformations, curvatures, and other geometric properties [6]. Coding schemes can either be descriptive or judgmental [6]. Descriptive coding schemes depend on surface properties and what the face can do to describe facial expressions. Judgmental coding schemes depend on the latent emotions or affects that produce them to parameterize facial expressions. The facial action coding system (FACS) [11] is one example of a descriptive system. The FACS is a system that describes all visually evident facial movements [11,12]. It divides facial expressions into individual components of muscle movement, called Action Units (AUs). Coding schemes such as facial AUs as well as geometric and/or appearance features can then be treated as input parameters to machine learning regressors or classifiers for the prediction of cognitive states. 

In the remainder of this paper, we will provide a review of the literature (Section 2), followed by a description of the methods used in our solution (Section 3). Then, we include a discussion of our results (Section 4). Finally, we will conclude this paper with some closing remarks (Section 5).

## 2. An Overview of the Literature

RECOLA [2] is a multimodal database of natural emotions that is often used in studies on the prediction of cognitive states. It contains video, audio, and physiological recordings. It also provides predesigned features for these recordings. Arousal and valence annotations were provided by six raters every 40 milliseconds of recording. The mean of the six ratings was used to label the data in our work. The database contains 5 min video recordings of 27 participants, where only data from 23 participants are publicly available. Since some of the data modalities in RECOLA contain records for 18 of the participants, we only used these 18 recordings from the RECOLA database to prove our concept. 

The authors of the original RECOLA database [2] further extended their work in [12], where they performed experiments on the database for the prediction of arousal and valence values. They extracted 20 visual features on each video frame in the video recordings of RECOLA along with their first-order derivates. They then deployed a bidirectional long short-term memory recurrent neural network (BiLSTM RNN) to predict arousal and valence measures. They compared the prediction performance of the RNN between mean ratings (average of annotations from all six raters) and all six ratings, using both single-task and multi-task learning techniques. For arousal, they achieved a CCC of 0.4270 using multi-task learning over all six ratings. For valence, they achieved a CCC of 0.4310 using single-task learning over all six ratings. The authors of RECOLA [2,12] later introduced the Audio/Visual Emotion Challenge and Workshop (AVEC) in 2015 [13]. 

In AVEC 2018 [14], the authors of RECOLA experimented with the different types of visual features: appearance, geometric, 17 facial AUs, and bags-of-words (BoWs). For arousal, they achieved a CCC of 0.3120 via multi-task Lasso, while using appearance features. For valence, they achieved a CCC of 0.4380 via a support vector machine (SVM), while using geometric features.

Other authors have also benefited from using the RECOLA database in their research. Han et al. [15] exploited the geometric visual features provided by AVEC to predict arousal and valence values through an RNN. They implemented an implicit fusion framework for joint audiovisual training. They achieved a CCC of 0.4130 and 0.5270 on arousal and valence predictions, respectively. Albadawy et al. [16] used the visual features provided by AVEC 2015, which included appearance (LGBP-TOP) and geometric (Euclidean distances between 49 facial landmarks) features. For arousal and valence predictions, they proposed a joint modelling strategy using a deep BiLSTM for ensemble and end-to-end models. Their ensemble BiLSTM model achieved a CCC of 0.6990 and 0.6170 for arousal and valence predictions from visual features, respectively. Weber et al. [17] used visual features provided by RECOLA’s team in 2016 to perform regression via an SVM with a late subject and multimodal fusion (at a decision/prediction level). Their best CCCs were 0.6820 and 0.4680 for arousal and valence, respectively.

Amirian et al. [18] used random forests to predict arousal and valence values from RECOLA’s audio, video, and physiological data. For visual features, they achieved a CCC of 0.5140 and 0.4980 on arousal and valence predictions, respectively. The End2You tool [19] is a toolkit for multimodal profiling that was developed by the Imperial College of London to perform continuous dimensional emotion labels of arousal and valence values. It can use raw videos as input. For RECOLA’s videos, it achieved a CCC of 0.3580 for arousal, and 0.5610 for valence. 

Brady et al. [20] used CNN features to predict arousal and valence values from video recordings using an RNN. They achieved an RMSE of 0.2010, a PCC of 0.4150, and a CCC of 0.3460 on arousal predictions. They achieved an RMSE of 0.1070, a PCC of 0.5490, and a CCC of 0.5110 on valence predictions. The authors of [21] exploited CNN features from RECOLA’s videos as well as an RNN to estimate valence values. They obtained an RMSE of 0.1070, a PCC of 0.5540, and a CCC of 0.5070.

In our work, we used and further processed the basic visual features extracted by the authors of RECOLA in [12] and experimented with a variety of regressors to predict the arousal and valence values of cognitive states.

## 3. Methods

In our work, 18 RECOLA videos were preprocessed. We processed the video recordings of RECOLA by applying frame extraction and sequencing, face detection and cropping, annotation labelling, and data augmentation. After processing, the extracted images (i.e., video frames) of participants’ full faces were inputted into the MobileNet-v2 and ResNet-18 CNNs for predicting arousal and valence values [4,5]. Since MobileNet-v2 performed better than the ResNet-18 CNN [4,5], we then used the trained MobileNet-v2 CNN to extract deep visual features. The extracted deep features were later used as input to classical regressors for predicting arousal and valence values. 

**Figure 1:**
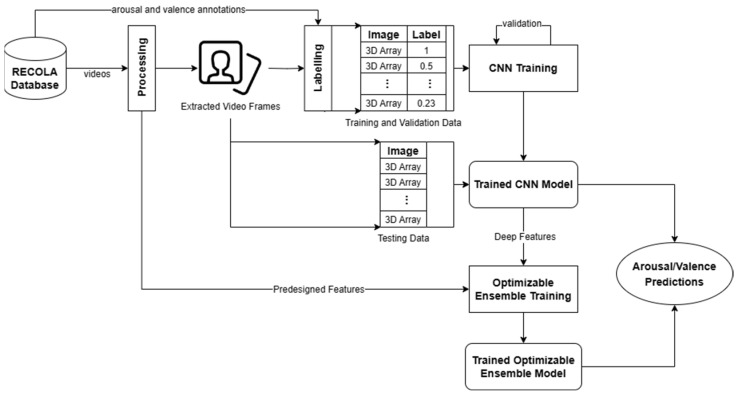
Overview of our visual data methodology.

We processed the visual features of RECOLA by applying time delay and sequencing, arousal and valence annotation labelling, and data shuffling and splitting. We then trained and tested classical regressors to predict the arousal and valence values. The following sections will discuss the details of our processing steps and regression methodology. Figure 1 shows an overview of our methodology for processing visual data as detailed in the following sections.

### 3.1. Processing of Video Recordings

The videos available in the RECOLA database are approximately 5 min long each. They were processed by extracting their video frames at a rate of 25 frames per second. As a result, we obtained an image frame every 40 milliseconds of video recording. That is a total of approximately 7500 frames per video. For data synchronization across all data modalities contained in the RECOLA database, we skipped the first 50 frames. For example, we would have acoustic, physiological, and visual samples collected at the 40th millisecond of recording, the 80th millisecond of recording, and so on. 

Face detection was then applied to narrow the prediction area [4,5]. We used the cascade object detector based on the Viola–Jones algorithm to detect people’s faces [22]. Following face detection, we noticed that the algorithm failed to detect faces in some of the obtained video frames. Hence, we cropped these images according to the face coordinates of the nearest image with a detected face. In the best-case scenario, the nearest image with a detected face would be the image preceding or following the image with a missed face. In the worst-case scenario, the algorithm would have failed to detect faces in a group of images, where the nearest image with a detected face would be more than one video frame away. In this case, the coordinates of the face might be off due to the movement of the participant in the video. Thus, manual intervention to edit the images was required.

We later cropped the images of faces to contain the lower half of the face (i.e., half of the nose, mouth, cheeks, and chin) for the purpose of VR applications, where head-mounted displays, covering the eyes and parts (or all) of the nose, are typically worn [5]. All face images were cropped by cutting off the upper half of the images automatically through MATLAB R2024a.

The data in RECOLA were labelled with respect to the arousal and valence emotional dimensions. The data samples were manually annotated using ANNEMO, an annotation tool developed by Ringeval et al. [2]. Each recording was annotated by six raters. The mean of these six ratings was used to label the data in our work. The mean arousal and valence values were also sampled every 40 milliseconds. The first 50 annotations (2 s × 25 samples per second) were ignored. The remaining annotations were accordingly used to label the corresponding visual samples. All labelling and fusion of data samples and features were carried out based on the recording times.

Data shuffling ensures the randomization and diversity of the data. The video frames were shuffled and split, where 80% went towards training and validation, and 20% went towards testing. Table 1 represents the breakdown of the extracted video frames.

### 3.2. Extraction and Processing of Predesigned and Deep Visual Features

In this section, we will discuss our methodologies for extracting and processing predesigned visual features, as well as deep visual features.

#### 3.2.1. Predesigned Visual Features

The video recordings of RECOLA were sampled at a sampling rate of 25 frames per second, where visual features were extracted for each video frame [12]. As predesigned visual features, RECOLA contains 20 attributes alongside their first-order derivative, resulting in 40 features in total. These attributes/features include 15 facial AUs of emotional expressions, the head pose in three dimensions (i.e., X, Y, Z), and the mean and standard deviation of the optical flow in the region around the head. The AUs are AU1 (Inner Brow Raiser), AU2 (Outer Brow Raiser), AU4 (Brow Lowerer), AU5 (Upper Lid Raiser), AU6 (Cheek Raiser), AU7 (Lid Tightener), AU9 (Nose Wrinkler), AU11 (Nasolabial Deepener), AU12 (Lip Corner Puller), AU15 (Lip Corner Depressor), AU17 (Chin Raiser), AU20 (Lip Stretcher), AU23 (Lip Tightener), AU24 (Lip Pressor), and AU25 (Lips Part) from the FACS. For more information about these features and their extraction, please refer to [12]. We used these features in our work on images of full faces extracted from the video recordings of RECOLA. For our work on half-face images for the purpose of VR applications, we only used the subset of AUs that pertains to the lower half of the face: AU6 (Cheek Raiser), AU11 (Nasolabial Deepener), AU12 (Lip Corner Puller), AU15 (Lip Corner Depressor), AU17 (Chin Raiser), AU20 (Lip Stretcher), AU23 (Lip Tightener), AU24 (Lip Pressor), and AU25 (Lips Part).

RECOLA’s video recordings were sampled at a rate of 25 frames per second. This means that 1 frame was captured every 0.04 s (40 milliseconds). The predesigned visual features were calculated on each frame, meaning that they were provided every 40 milliseconds as well. Since other data modalities of RECOLA only started being recorded after 2 s (2000 milliseconds), we skipped any readings that occurred before that time. As a result, the first 50 frames (2 s × 25 frames per second) of the recordings were unused in our work.

As we proceeded in Section 3.1, the first 50 annotations were discarded. The remaining annotations were accordingly used to label the corresponding vectors of visual features. All the labelling and fusion of data samples and features were completed according to the timing of the video frames.

We also shuffled the processed data samples to randomize the data. The collected video frames were then split using an 80–20% split for validation and testing. Our training and validation dataset of predesigned visual features was 106,201 frames × 40 features in size, while the testing dataset was 26,550 frames × 40 features in size.

#### 3.2.2. Deep Visual Features

We previously trained CNNs such as ResNet-18 and MobileNet-v2 on the video frames of RECOLA [4,5]. After performing multiple test scenarios, we observed that MobileNet-v2 outperformed ResNet-18. To identify if it is possible to further improve the performance we achieved in [4,5], we extracted deep visual features through our trained MobileNet-v2. These features have been extracted to replace or to be fused with the predesigned visual features from Section 3.2.1. To the best of the authors’ knowledge, this combination of predesigned and deep visual features is unique as it was not attempted by other researchers who are using the RECOLA database in their studies.

After training the MobileNet-v2 CNN on predicting arousal and valence values from images of full/half faces, we used the trained network(s) to extract deep visual features from the input images. The deeper layers of the network contain higher-level features that are constructed using the lower-level features from earlier layers. To extract the features of the training and testing images, we used activations on the global pooling layer at the end of the network. The global pooling layer pooled features over all spatial locations, providing 1280 features in total. Table 2 summarizes the dimensions of the extracted sets of the deep visual features.

In an attempt to further improve the prediction performance, we further fused the deep visual features with the predesigned visual features. Adding more features as the input to machine learning regressors boosts their performance since this provides more descriptive information about the data. As a result, we obtained feature sets of 1320 features. Table 3 shows a breakdown of the resulting feature sets of predesigned and deep visual features for images of full/half faces.

### 3.3. Regression

In this section, we will discuss our machine learning regression methodologies for predicting arousal and valence values from the extracted video frames and predesigned/deep visual features. 

#### 3.3.1. Deep Machine Learning

As mentioned previously, we experimented with two pretrained MATLAB CNNs: ResNet-18 and MobileNet-v2 in [4,5]. To fine-tune the pretrained CNNs for regression to predict arousal and valence values, we customized the layers of each CNN to suit our needs and applied data augmentation. We, thus, replaced the image input layer to make it accept images of size 280 × 280 × 3. Additionally, we replaced the final fully connected layer and the classification output layer with a fully connected layer of size 1 (the number of responses, i.e., the arousal/valence value) and a regression layer. The convolutional layers of the CNNs extract image features that are then used by the last learnable layer and the final classification layer to classify the input image [23]. These layers have information about converting the extracted features into class probabilities, loss values, and predicted labels. In the cases of ResNet-18 and MobileNet-v2, the last learnable layer is the fully connected layer. We adjusted the learning rates of the last learnable layer in order to make the CNNs learn faster in the new fully connected layer than in the transferred/pretrained convolutional layers by setting the learning rate factors for weights and biases to 10.

The amount of training data was increased by applying randomized data augmentation. Data augmentation allows CNNs to train to be invariant to distortions in image data and helps to prevent overfitting by preventing the CNN from memorizing the exact characteristics of training images. We use augmentation options such as random reflection in the *x*-axis, random rotation, and random rescaling. As mentioned before, we replaced the image input layer of the pretrained CNNs (ResNet-18 and MobileNet-v2) to allow them to take larger input images of size 280 × 280 × 3, but the images in our video frames did not all have this size. Therefore, we used an augmented image datastore to automatically resize the images. We also specified additional augmentation operations to perform on the images in order to prevent the CNNs from memorizing image features. We randomly reflected the images along the vertical *x*-axis, randomly rotated them from the range [–90, 90] degrees, and randomly rescaled them from the range [1, 2]. These changes do not affect the contents of the training images; however, they will help the CNNs in extracting/learning more features from the images.

We modified the training options and parameters depending on the size of our input data. Table 4 summarizes the training parameters we used for training the CNNs. We experimentally set the initial learning rate to 0.0001 and the number of epochs to 30. As there were 84,960 training images, we set the minimum batch size to 9 in order to evenly divide the training data into 9440 equal batches and ensure that the whole training set was used during each epoch. This resulted in 9440 iterations per epoch (84,960/9 = 9440). For validation frequency, we divided the number of iterations by 2 to ensure that the training process was validated at least twice per training epoch. We used the stochastic gradient descent with momentum (SGDM) optimizer for training.

#### 3.3.2. Classical Machine Learning

For the prediction of arousal and valence values, we then used the extracted sets of predesigned and/or deep visual features to train, validate, and test an optimizable ensemble regressor. An optimizable regression ensemble optimizes training hyperparameters (ensemble method, the number of learners, learning rate, minimum leaf size, and the number of predictors to sample) via Bayesian optimization. The optimizable ensemble regressor, trained on visual features, used the LSBoost algorithm with Bayesian optimization to obtain the best prediction performance. We implemented a 5-fold cross-validation during training to avoid overfitting.

### 3.4. Decision Fusion

We fused the testing predictions from the optimizable ensembles and MobileNet-v2 by averaging them to observe how this fusion affected the prediction performance. Let N be the number of trained models and P be the predictions set obtained by model i; the final predictions set, $P_{final}$, can then be computed as follows:

(1) $$P_{final}=\frac{P_{1}+P_{2}+\cdots +P_{n}}{N}=\frac{\sum_{i=1}^{n}P_{i}}{N}$$

## 4. Discussion of Results

After training the MobileNet-v2 CNN and optimizable ensemble models, we tested them by predicting the arousal and valence values on the testing sets to evaluate the performance when presented with new data. Table 5 summarizes the validation and testing performances in terms of the RMSE, PCC, and CCC performance measures. A smaller RMSE value signifies better performance, whereas greater PCC and CCC values signify better performance. 

While using the deep visual features set from full-face images, we have achieved a testing RMSE of 0.1204, a PCC of 0.7707, and a CCC of 0.7640 on arousal predictions. We achieved a testing RMSE of 0.0812, a PCC of 0.7761, and a CCC of 0.7530 on valence predictions. While using the combined (predesigned and deep) visual features set from full-face images, we achieved a testing RMSE of 0.1098, a PCC of 0.8138, and a CCC of 0.7974 on arousal predictions, respectively. We achieved a testing RMSE of 0.0784, a PCC of 0.7947, and a CCC of 0.7834 on valence predictions, respectively. We further used images of half faces since we aimed to integrate our solution into a practical VR application using head-mounted displays, which cover the top half of the face. For half-face images, we only trained the optimizable ensemble model on the combined (predesigned and deep) visual features set. As a result, we achieved a testing RMSE of 0.1187, a PCC of 0.7780, and a CCC of 0.7505 on arousal predictions and an RMSE of 0.0832, a PCC of 0.7633, and a CCC of 0.7360 on valence predictions. As can be seen, the use of half-face images for feature extraction has impacted the prediction performance negatively. This is because fewer visual features can be identified from smaller images containing partial facial features (i.e., missing the eyes).

Table 5 also compares our results with other results from the literature. As shown in Table 5, our performances are better than those from the literature [12,14,15,16,17,18,19,20,21] which performed more complex processing and feature extraction. Our prediction performances remained better even when we operated on images of the lower half of the face, as compared to others who used features from images of whole faces.

In Table 5, the validation performances were evaluated by performing a 5-fold cross-validation across the training data. The testing performances were computed by using the trained model for predicting the arousal and valence values of the testing set. Table 6 also compares the performances of our models for learned [4,5], predesigned features [7], deep features, and combined features. 

**Figure 2:**
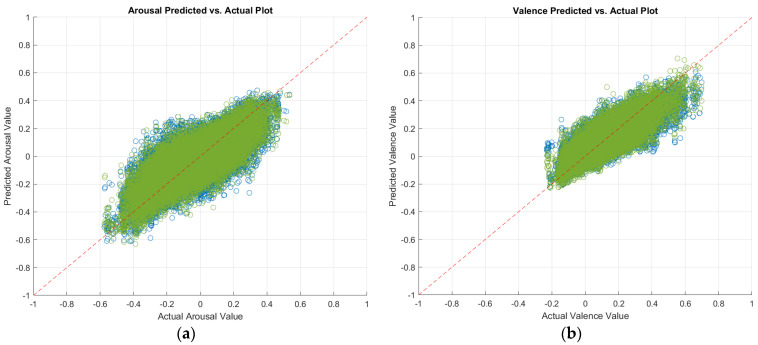
Predicted versus actual plots of fused (**a**) arousal, and (**b**) valence predictions from an optimizable ensemble trained on combined visual features and MobileNet-v2 trained on video frames of full faces (green) or half faces (blue). The red dashed line represents perfect predictions.

In an attempt to further improve our prediction performances, we also fused the predictions of the trained optimizable ensemble regressors and MobileNet-v2 CNNs as described in Section 3.4. Namely, we fused the predictions of an optimizable ensemble trained on combined (predesigned and deep) visual features and the predictions of the MobileNet-v2 trained on images of full/half faces. Table 6 shows the prediction performances that we achieved after decision fusion. As shown in the table, decision fusion has improved our prediction performances. Figure 2 displays a plot of the predicted arousal and valence values against the actual values after decision fusion. In the plot of a perfect regression model, the predicted values would be the same as the actual values, resulting in a diagonal line of points [23]. Models where the points are scattered near the diagonal line represent good models, with fewer errors. 

## 5. Conclusions

In conclusion, we performed arousal and valence predictions by exploiting learned, predesigned, and deep visual features from the video recordings of the RECOLA database. We aimed to determine the best model(s) to be integrated into a VR system that runs cognitive remediation exercises for users with mental health disorders such as schizophrenia. The learned method was performed using a MobileNet-v2 CNN trained on images of full/half faces. The predesigned features vector was provided in and extracted from the RECOLA database. The deep features vector was extracted from the trained MobileNet-v2. The feature vectors were processed and accordingly labelled with their corresponding arousal or valence annotations. We trained, validated, and tested an optimizable ensemble regressor to predict arousal and valence values. We also tested the optimizable ensemble model using a combination of deep and predesigned features. To the best of the authors’ knowledge, our prediction performances on arousal and valence predictions are higher in comparison to the literature. To further improve our prediction performance, we performed decision fusion using the predictions of the different models that we have tested. After applying decision fusion on predictions obtained by an optimizable ensemble trained on combined (predesigned and deep) visual features and a MobileNet-v2 CNN trained on video frames of half faces, we achieved an RMSE of 0.1140, a PCC of 0.8000, and a CCC of 0.7868 on arousal predictions. For valence, we achieved an RMSE of 0.0790, a PCC of 0.7904, and a CCC of 0.7645. Since we achieved good prediction performance using physiological [3,4] and visual data, we will work on acoustic data. We can later combine our solutions for the different data modalities. 

One limitation of this study is the lack of real data in the context of VR applications. We have been using the RECOLA database as a proof of concept. In the future, researchers can apply our findings to real data obtained from a VR system. In addition, future studies could explore the use of other sensors to not only predict emotional states but also measure cognitive effort during VR immersions to enhance the treatments for individuals with mental health disorders.

## Figures and Tables

**Table 1:** Breakdown of video frames.

Parameters	Original	80–20% Split
Training Frames	106,201	84,960
Validation Frames	N/A	21,241
Testing Frames	26,550	26,550
Total	132,751	132,751

N/A (Not Applicable).

**Table 2:** Dimensions of extracted sets of deep visual features.

Dataset	Number of Face Images	Deep Visual Features	Final Dimensions
Original	132,751	1280	132,751 × 1280
Training	106,201	1280	106,201 × 1280
Testing	26,550	1280	26,550 × 1280

**Table 3:** Breakdown of predesigned and deep visual feature sets.

Dataset	Number of Face Images	Predesigned Visual Features (Full-Face Images)	Predesigned Visual Features (Half-Face Images)	Deep Visual Features	Final Dimensions
Original	132,751	40	9	1280	132,751 × 1320 or 132,751 × 1289
Training	106,201	40	9	1280	106,201 × 1320 or 106,201 × 1289
Testing	26,550	40	9	1280	26,550 × 1320 or 26,550 × 1289

**Table 4:** CNN training parameters.

Parameters and Options	Original	80–20% Split
Training Images	106,201	84,960
Validation Images	N/A	21,241
Testing Images	26,550	26,550
Learning Rate	0.0001	
Minimum Batch Size	9	
Number of Epochs	30	
Iterations per Epoch	84,960/9 = 9440	
Validation Frequency	9440/2 = 4720	
Optimizer/Learner	SGDM	

N/A (Not Applicable).

**Table 5:** Summary of prediction performances.

Prediction	Data	Regression Model	Validation RMSE	Testing RMSE, PCC, CCC
Arousal	Deep Visual Features (Full Faces)	Optimizable Ensemble	0.1196	0.1204, 0.7707, 0.7640
	Predesigned Visual Features (Full Faces)	Optimizable Ensemble [7]	0.1079	0.1033, 0.8498, 0.8001
	Deep and Predesigned Visual Features (Full Faces)	Optimizable Ensemble	0.1096	0.1098, 0.8138, 0.7974
	Deep and Predesigned Visual Features (Half Faces)	Optimizable Ensemble	0.1183	0.1187, 0.7780, 0.7505
	Images of Full Faces	MobileNet-v2 [4]	0.1218	0.1220, 0.7838, 0.7770
	Images of Half Faces	MobileNet-v2 [5]	0.1257	0.1259, 0.7761, 0.7717
	Predesigned Visual Features (Full Faces)	Single-Task RNN [12]	N/A	N/A, N/A, 0.4270
	Predesigned Visual Features (Full Faces)	Multi-Task Lasso [14]	N/A	N/A, N/A, 0.3120
	Predesigned Visual Features (Full Faces)	RNN [15]	N/A	N/A, N/A, 0.4130
	Predesigned Visual Features (Full Faces)	BiLSTM RNN [16]	N/A	N/A, N/A, 0.6990
	Predesigned Visual Features (Full Faces)	SVM [17]	N/A	N/A, N/A, 0.6820
	Predesigned Visual Features (Full Faces)	Random Forests [18]	N/A	N/A, N/A, 0.5140
	Raw Videos	End2You Tool [19]	N/A	N/A, N/A, 0.3580
	Deep Visual Features (Full Faces)	RNN [20]	N/A	0.2010, 0.4150, 0.3460
Valence	Deep Visual Features (Full Faces)	Optimizable Ensemble	0.0818	0.0812, 0.7761, 0.7530
	Predesigned Visual Features (Full Faces)	Optimizable Ensemble [7]	0.0733	0.0702, 0.8473, 0.8053
	Deep and Predesigned Visual Features (Full Faces)	Optimizable Ensemble	0.0798	0.0784, 0.7947, 0.7834
	Deep and Predesigned Visual Features (Half Faces)	Optimizable Ensemble	0.0837	0.0832, 0.7633, 0.7360
	Images of Full Faces	MobileNet-v2- [4]	0.0831	0.0823, 0.7789, 0.7715
	Images of Half Faces	MobileNet-v2 [5]	0.0848	0.0840, 0.7645, 0.7510
	Predesigned Visual Features (Full Faces)	Single-Task RNN [12]	N/A	N/A, N/A, 0.4310
	Predesigned Visual Features (Full Faces)	SVM [14]	N/A	N/A, N/A, 0.4380
	Predesigned Visual Features (Full Faces)	RNN [15]	N/A	N/A, N/A, 0.5270
	Predesigned Visual Features (Full Faces)	BiLSTM RNN [16]	N/A	N/A, N/A, 0.6170
	Predesigned Visual Features (Full Faces)	SVM [17]	N/A	N/A, N/A, 0.4680
	Predesigned Visual Features (Full Faces)	Random Forests [18]	N/A	N/A, N/A, 0.4980
	Raw Videos	End2You Tool [19]	N/A	N/A, N/A, 0.5610
	Deep Visual Features (Full Faces)	RNN [20]	N/A	0.1070, 0.5490, 0.5110
	Deep Visual Features (Full Faces)	RNN [21]	N/A	0.1070, 0.5540, 0.5070

N/A (Not Applicable).

**Table 6:** Decision fusion prediction performances.

Prediction	Data	Fused Models	Testing RMSE, PCC, CCC
Arousal	Deep and Predesigned Visual Features and Images of Full Faces (Learned)	Optimizable Ensemble and MobileNet-v2	0.1069, 0.8264, 0.8130
	Deep and Predesigned Visual Features and Images of Half Faces (Learned)	Optimizable Ensemble and MobileNet-v2	0.1140, 0.8000, 0.7868
Valence	Deep and Predesigned Visual Features and Images of Full Faces (Learned)	Optimizable Ensemble and MobileNet-v2	0.0742, 0.8181, 0.8040
	Deep and Predesigned Visual Features and Images of Half Faces (Learned)	Optimizable Ensemble and MobileNet-v2	0.0790, 0.7904, 0.7645

## Data Availability

No new data were created. Data was obtained from the RECOLA team and are available, upon request at https://diuf.unifr.ch/main/diva/recola/download.html, accessed on 28 May 2024.
